# Orchestrating infection: the impact of RyfA and TimR sRNAs on stress resistance and virulence in avian pathogenic *Escherichia coli* in chickens

**DOI:** 10.1128/aem.02000-25

**Published:** 2026-04-29

**Authors:** Carole Anamalé, Hicham Bessaiah, Evelyne Ng Kwan Lim, Sébastien Houle, Éric Massé, Charles M. Dozois

**Affiliations:** 1 Institut National de la Recherche Scientifique, Centre Armand-Frappier Santé Biotechnologie 14850 , Laval, Québec, Canada; 2 EVAH Corp – Evolution for Animal Health, Laval, Québec, Canada; 3 Centre de Recherche en Infectiologie Porcine et Avicole (CRIPA), Faculté de Médecine Vétérinaire, Université de Montréal Saint-Hyacinthe 70354 , Saint-Hyacinthe, Québec, Canada; 4 Department of Biochemistry, RNA Group, Université de Sherbrooke 7321, Sherbrooke, Québec, Canada; Universidad de los Andes, Bogotá, Colombia

**Keywords:** stress response, virulence, TimR, RyfA, sRNA, APEC/ExPEC

## Abstract

Avian pathogenic *Escherichia coli* (APEC) strains collectively represent a subpathotype of extraintestinal pathogenic *E. coli* (ExPEC) and are associated with substantial economic losses to the poultry industry, amounting to hundreds of millions of dollars worldwide. While chickens are the primary host, APEC strains also pose a zoonotic threat through cross-host transmission via contaminated meat or eggs. Supporting this, APEC shares numerous virulence factors and genetic features with uropathogenic *E. coli* (UPEC), another well-characterized ExPEC subpathotype. RyfA, a small regulatory RNA (sRNA), has been identified in UPEC as an essential regulator in stress resistance and virulence. However, its role in APEC, as well as that of another sRNA, TimR, located in the same genomic region, remains unclear. In this study, *ryfA* deletion in the APEC O1:K1 strain CH138 significantly reduced virulence in both single-strain and competitive co-infection models in chickens. Loss of *ryfA* decreased type 1 fimbria production (pili) *in vitro*, increased sensitivity to osmotic and oxidative stress, and impaired APEC internalization and survival within macrophages. In contrast, CH138 Δ*timR* and ΔΔ*ryfA-timR* mutants exhibited phenotypes similar to the wild-type strain. These findings suggest RyfA is required for stress adaptation in APEC strain CH138, likely contributing to virulence and survival during infection. The absence of additive effects in the double mutant suggests that attenuation of the *ryfA* mutant is intrinsically linked to effects caused by TimR in the absence of RyfA. Elucidating the regulatory interplay between RyfA and TimR will advance understanding of APEC pathogenesis and may highlight conserved sRNA targets for novel therapeutic approaches.

**IMPORTANCE:**

Avian pathogenic *Escherichia coli* (APEC) strains cause colibacillosis in poultry, leading to significant financial losses and representing a potential zoonotic reservoir. Understanding how APEC persists in the host and withstands environmental stress is crucial for animal and public health. We identify the small regulatory RNA (sRNA), RyfA, as a key determinant of APEC virulence. Loss of RyfA impairs stress resistance, reduces type 1 fimbriae, limits survival in macrophages, and attenuates infection in chickens. By contrast, loss of the adjacent sRNA TimR had no discernible phenotypic effects compared to the wild-type parent. Interestingly, the combined loss of both *ryfA-timR* abrogated any phenotypic effects on virulence or stress resistance, suggesting the potential interplay between these sRNAs in gene regulation. These results demonstrate the critical role of RyfA for APEC pathogenesis and suggest that conserved sRNA-mediated regulatory pathways could provide future targets for prevention or treatment to improve poultry health and food safety.

## INTRODUCTION

Avian pathogenic *Escherichia coli* (APEC) strains belong to the extraintestinal pathogenic *E. coli* (ExPEC) pathotype and exhibit substantial genetic and virulence-related similarities with other ExPEC pathotypes, such as uropathogenic *E. coli* (UPEC) and neonatal meningitis *E. coli* (NMEC), which are responsible for urinary tract infections and meningitis in humans, respectively (1–3). APEC strains cause extraintestinal disease in avian species, including turkeys, ducks, and chickens. The most prevalent and economically impactful disease in poultry is respiratory tract infection, commonly referred to as colibacillosis, which is characterized by a complex of respiratory and systemic illnesses. Colibacillosis affects chickens across all age groups, including layers and broilers. Beyond being a major animal health concern, colibacillosis induced by APEC represents a significant animal welfare issue and contributes to substantial economic losses in poultry production worldwide, amounting to hundreds of millions of dollars annually, including $40 million per year in the United States (4, 5). These economic losses are primarily attributed to decreased egg and meat production, increased mortality rates, decreased hatching rates and live weight, and elevated rates of carcass condemnation at slaughterhouses (6). Commonly reported APEC serogroups include O1, O2, O78, O35, and O145 (4, 7, 8). Due to potential zoonotic risks, APEC causes diseases critical to the poultry industry, and such strains from poultry are also a concern to public health (1, 9, 10).

Several potential virulence factors have been associated with APEC, including the aerobactin iron-sequestering system, K1 capsular antigen, temperature-sensitive hemagglutinin (Tsh), resistance to the bactericidal effects of serum, and adhesins such as curli, P fimbriae, and type 1 fimbriae (pili) (4, 11). Adhesion can be considered the primary and most crucial step for bacterial infection. Some studies have demonstrated the importance of type 1 fimbriae in APEC strains to adhere to chicken epithelial cells in the pharynx and trachea, thereby facilitating invasion (12–14).

Although type 1 fimbriae appear to play a less prominent role in APEC compared to UPEC strains, APEC strains with mutations that decrease the expression of type 1 fimbriae have been shown to be attenuated (15–17), and type 1 fimbriae can contribute to both adherence and cellular invasion by APEC (14, 18, 19). Expression of type 1 fimbriae is under the control of the *fim* operon*,* which is governed by a phase-variable element, meaning that the promoter located within an invertible element (IE) *fimS* can switch between two different orientations, ON or OFF phase. The expression of type 1 fimbriae is tightly regulated by a complex network of both global and specific regulators. Several studies have highlighted the connection between environmental stress and the regulation of type 1 fimbriae (20, 21). Building on these findings, as well as our own results (14, 15, 22), we propose that since type 1 fimbriae play a crucial role in ExPEC colonization and virulence, it is important to understand how stress response pathways influence their expression. Accordingly, we hypothesize that genes affecting the regulation of type 1 fimbria expression may also be involved in broader regulatory networks governing bacterial stress adaptation.

In recent years, small bacterial regulatory RNAs (sRNAs) have emerged as key post-transcriptional regulators of numerous physiological and virulence-related processes (23–26). Several studies have demonstrated the crucial role of sRNAs in bacterial adaptation and stress tolerance, including envelope stress (27), osmotic stress, and oxidative stress, as illustrated with the well-characterized sRNA OxyS (28). sRNAs linked to nutrient limitation, such as low phosphate conditions (EsrL) (29) and iron deficiency with RyhB (30, 31), also play an important role in cellular homeostasis and virulence. Recently, the sRNA RyfA has been implicated in multiple processes, including pathogenesis, motility, and biofilm formation in various pathotypes of *E. coli* (15, 32). While the role of RyfA has been studied in UPEC, its contribution to virulence in APEC, in addition to the combined influence of a second sRNA adjacent to RyfA, TimR, has not been investigated (see Fig. S7 for the genetic organization of the region encoding these sRNAs). Given that APEC strains are a major cause of extraintestinal infections in poultry and share genetic and virulence-related similarities with human ExPEC strains (33), it is essential to elucidate whether regulatory mechanisms underlying ExPEC virulence are conserved and required for extra-intestinal infection in avian as well as human hosts.

Based on previous evidence supporting the importance of RyfA in general stress response, this study aimed to investigate the role of RyfA in the APEC strain CH138. Notably, a newly identified sRNA, TimR, was identified in the same genomic region as RyfA in *Salmonella enterica* serovar Typhimurium (34), and a similar region is also present in *E. coli* strains (15) (Fig. S7). In our study, we investigated the roles of RyfA, TimR, or both sRNAs in the APEC strain CH138 for virulence in a chicken respiratory infection model and for adaptations to various stress conditions, including survival in macrophage cells, resistance to osmotic and oxidative stress, and serum resistance.

## MATERIALS AND METHODS

### Animals

All chickens used in this study were clinically healthy. White Leghorn specific-pathogen-free (SPF) chickens were acquired from the Canadian Food Inspection Agency (CFIA) (Ottawa, ON). When received, birds were 1 or 2 days old and were acclimated for 7 days prior to experimentation. Animals were infected at 3 weeks of age. The study for the chicken experiment lasted for 2 days (48 h). Euthanasia was performed using isoflurane, followed by CO_2_ with a maximum of 10 L per minute (LPM). After approximately 3–4 min, the animal’s breathing was checked to confirm cessation.

### Bacterial strains, growth conditions, and plasmids

*E. coli* strains and plasmids used in this study are listed in Table S1. *E. coli* CH138 (O1:K1:H7), ST95, was initially isolated from a chicken infected with colibacillosis in Quebec (35). Bacteria were maintained as glycerol stocks at −80°C ± 10°C and were grown in Lysogeny Broth (LB) (Alpha Bioscience, Baltimore, MD) at 37°C. Antibiotics and reagents were added as required at the following concentrations: kanamycin, 50 μg/mL (Bioshop Canada Inc., Burlington, Ontario KAN201); ampicillin, 100 μg/mL (Bioshop Canada Inc., AMP201); and chloramphenicol, 30 μg/mL (Bioshop Canada Inc., CLR201).

### Construction of site-directed mutants and complementation of strains

Mutant strains were constructed using the λ-Red recombination procedure established by Datsenko and Wanner, with plasmid pKD4 as the template for the kanamycin resistance cassette (36). Briefly, the CFT073 strain expressing λ-Red recombinase from plasmid pKD46 was created from PCR products that contained the kanamycin resistance cassette flanked by regions homologous to the target gene (*ryfA*, *timR,* or *ryfA-timR*). After selecting recombinants on plates containing kanamycin, PCR was used to confirm gene deletions. The primers used for gene disruption are listed in Table S2. In mutant strains, antibiotic resistance cassettes flanked by FLP recombination target (FRT) sequences were excised by introducing pCP20, which expresses the FLP recombinase (37).

For complementation, the mutant strains were complemented by inserting the respective genes at the *att*Tn*7* chromosomal site. This procedure was carried out as described by Crépin et al. (38). Briefly, the target gene (*ryfA*), along with its native promoter, was cloned into the vector pGP-Tn7-Cm and conjugated into the strain that carried the transposase plasmid pSTNSK. The *ryfA*-complemented strain was produced after integration at the *att*Tn7 site, and conjugants were chosen on chloramphenicol plates and verified by PCR.

### Evaluation of type 1 fimbria production mediated by yeast agglutination

The level of type 1 fimbria production was assessed phenotypically by yeast agglutination as previously described (39). Bacterial strains were incubated in LB broth at 37°C until OD_600_ 0.6 ± 0.02 with shaking at 250 rpm. Briefly, after centrifugation, the pellets were resuspended in phosphate-buffered saline (PBS 1×, pH 7.4) to an initial concentration of approximately 2 × 10^11^ cells/mL. Then, in round-bottom 96-well plates, 40 µL of diluted samples were transferred and serially diluted 2-fold in equal volumes of 3% commercial yeast suspension. Following 30 min of incubation on ice, yeast agglutination was visualized. The agglutination titer was defined as the most diluted bacterial sample giving a positive aggregation reaction. The Δ*fim* type 1 fimbria-negative mutant strain was used as a negative control, and a mutant strain having a *fim*-locked ON promoter orientation was used as a positive control.

### Swimming motility assay

Motility assays to determine bacterial spreading due to flagella-mediated swimming motility were done using strain CH138 and its derivative mutants, following a protocol from a previous study (40) with some modifications. Bacterial strains were grown until mid-log exponential phase (OD_600_ 0.6 ±0.02). Then, with a sterile inoculating loop, the cultures were stabbed into the center of soft agar plates (1% tryptone, 0.5% NaCl, and 0.25% agar), which had been prepared 1 day in advance and allowed to set at room temperature overnight. Care was taken during inoculating to ensure that the bottom of the plate was not touched, preventing potential twitching motility. Plates were incubated at 37°C for 16, 20, and 24 h (±2 h), and motility diameters were measured for each strain at different time points.

### Biofilm formation assay

Biofilm assays were conducted using 96-well plates according to a previously described procedure (41). Briefly, strains were grown in LB medium at 37°C ± 2°C until either the mid-log or the stationary phase. Then, 200 μL of bacterial culture was added to each well, and the samples were incubated statically at 37°C for 48 h. After incubation, the wells were washed twice with PBS and stained with a 0.1% crystal violet solution (Sigma-Aldrich, Steinheim, Germany, 229288) for 30 min. Any unbound dye was removed by washing the plates four times with PBS. Once the plates were air-dried, a solution of acetone and ethanol (in an 80:20 volume ratio) was added to the wells. The biofilm density was quantified by measuring the absorbance at OD_595nm_.

### Growth experiments under conditions of osmotic stress

Wild-type APEC strain CH138 and derivative mutants, Δ*ryfA*, Δ*timR,* and ΔΔ*ryfA-timR,* were assessed for their ability to grow under osmotic stress induced by 0.6M urea (Roche Diagnostics, Mannheim, Germany, 1685902). From an overnight pre-culture grown in LB medium, bacteria were inoculated at a 1:100 ratio and then incubated at 37°C with shaking at 250 rpm until the mid-log exponential phase at OD_600_.

For assays on solid agar plates, when the OD was reached, strains were serially diluted and plated on LB agar alone as a control or LB agar supplemented with 0.6 M urea. After 16 h ± 2 h incubation at 37°C, colonies were counted, and growth under osmotic conditions was compared to growth on LB agar.

For assays in liquid, from mid-log exponential cultures, growth was determined using the automated turbidimetric “Bioscreen system” (Oy Growth Curves Ab Ltd., Raisio, Finland). Briefly, 2 µL of the culture was inoculated in 200 µL of LB medium supplemented with 0.6 M urea. The microtiter plates were incubated at 37°C, and optical densities (OD) were measured at regular intervals using the instrument’s wideband filter (600 nm) over 24 h. Prior to each measurement, the plates were agitated for 10 s at medium amplitude. OD readings were taken at 15-min intervals. These assays were repeated in two independent experiments, with 10 replicate wells per strain. Growth under osmotic conditions was compared to growth in LB liquid.

### Hydrogen peroxide sensitivity assay

The susceptibility to oxidative stress-inducing agents was assessed using an agar overlay diffusion method on LB plates (1.5% agar), following the protocol described by Sabri et al. (42). Bacterial strains were grown in LB at 37°C with shaking at 250 rpm until mid-log exponential phase at OD_600_ (0.6 ± 0.02). Then, 100 μL of each culture was mixed with 3 mL molten top agar (0.5% agar) and poured onto an LB agar plate; 7-mm diameter Whatman filter disks were placed in the center of the solidified overlays and then were dampened with 10 μL of 30% hydrogen peroxide (H_2_O_2_) (Sigma-Aldrich, 516813). The plates were then incubated for 16–18 h at 37°C. Following growth, the diameters of inhibition zones were measured.

### Propagation of monocyte/macrophage cell lines

The MQ-NCSU mononuclear cell line, established from the spleen of a broiler-type chicken experimentally challenged with the JM/102W strain of Marek’s disease virus (43), was used. Cells were cultured in RPMI-1640 (Sigma-Aldrich) supplemented with 7.5% heat-inactivated FBS (Sigma-Aldrich), 2.5% inactivated chicken serum (Sigma-Aldrich), and 2 mM L-glutamine, at 40°C in a 5% (vol/vol) CO_2_ incubator.

The human monocyte cell line THP-1 (ATCC TIB-202) was derived from the peripheral blood of a patient with acute monocytic leukemia. THP-1 cells were maintained in RPMI 1640 (Wisent, Saint-Jean-Baptiste, QC, Canada) containing 10% (vol/vol) heat-inactivated FBS (Wisent, Saint-Jean-Baptiste, QC, Canada), 1 mM sodium pyruvate (Wisent, Saint-Jean-Baptiste, QC, Canada), and 1% modified Eagle’s medium with non-essential amino acids (Wisent). A stock culture was maintained as monocyte-like, non-adherent cells at 37°C in an atmosphere containing 5% (vol/vol) CO_2_.

### *E. coli* intracellular survival assay

Internalized *E. coli* surviving within macrophages was enumerated by the standard gentamicin protection assay (44). For avian macrophages, MQ-NCSU cells (5 × 10^5^) were cultured in 24-well flat-bottomed plates for 24 h before the experiment. Prior to infection, THP-1 cells were differentiated by treating them with 25 nM phorbol 12-myristate 13-acetate (PMA) for 48 h and plated (5 × 10^5^) in 24-well flat-bottomed plates. Briefly, for both cell lines, bacteria were grown overnight in LB medium and then adjusted to an OD_600_ of 0.6 ± 0.02. The phagocytic cells were infected with each strain at a 20:1 multiplicity of infection (MOI). Plates were centrifuged for 5 min at 800 x *g* to synchronize bacterial uptake and incubated at 40°C ± 2°C for MQ-NCSU cells and 37°C ± 2°C for THP-1 cells in a 5% CO_2_-humidified air atmosphere for 1 h. After 1 h, extracellular bacteria were removed by washing the cells three times with phosphate-buffered saline (pH 7.4) (PBS). The cells were then either lysed with 0.1% PBS-DOC (T0) for 5 min at room temperature or incubated for an additional 2 h in fresh complete RPMI containing 100 µg/mL of gentamicin (T2) to kill extracellular bacteria and assess surviving bacteria. After another 2 h and three washes with PBS, the remaining cells were incubated for 24 h with 12 µg/mL of gentamicin (T24). Infected cells were subsequently harvested at the following four time intervals: 0, 2, 6, and 24 h post-infection (hpi). Following a PBS wash and lysis, bacteria were serially diluted, and surviving bacteria were enumerated by colony count (CFU) on LB agar plates. The results are expressed as the mean ± SEM of at least three experiments performed in triplicate. The nonparametric one-way ANOVA test was used for statistical analysis.

### Experimental infection of chickens via the air sacs

The abilities of different strains to disseminate in the respiratory tract and internal organs of chickens were compared using two different infection models. For both models, 3-week-old White Leghorn specific-pathogen-free (SPF) chickens were used. For the single-strain infection model, two independent experiments were performed, and the first 12 animals per group were used for CH138 O1:K1:H7 wild-type, the Δ*ryfA* mutant, and the *ryfA* complemented strain. In the second, 12 animals per group were also used for the CH138 O1:K1:H7 wild-type strain, the Δ*ryfA* mutant, the Δ*timR* mutant, and the ΔΔ*ryfA-timR* double mutant strain. All chickens per group were randomly reared in separate isolator cages with food and water available *ad libitum*. Each group was inoculated in the right thoracic air sac with 0.1 mL (10^6^ CFU) of a bacterial inoculum consisting of a diluted 24-h beef heart infusion broth culture of either *E. coli* CH138 O1:K1:H7 wild-type strain, the Δ*ryfA* mutant, the Δ*timR* mutant, or the ΔΔ*ryfA-timR* double mutant strain. For the competitive coinfection model, two independent experiments were performed with 12 and 15 animals per group, respectively. Preparations of strains were identical to those for the single-strain infections. Briefly, chickens were inoculated simultaneously with the *E. coli* CH138 O1:K1:H7 Δ*lac* and its isogenic mutants (Δ*ryfA,* Δ*timR,* or the ΔΔ*ryfA-timR* double mutant) with equal quantities (ranging from 3.00 to 6.00 × 10^6^ CFU) of each mutant strain and the *E. coli* CH138 Δ*lac* (42). After infection, blood samples were collected aseptically from each chicken at 6, 24, and 48 h following bacterial inoculation and were diluted in phosphate-buffered saline (pH 7.4). A 0.1 mL was plated on MacConkey agar plates. All surviving birds were euthanized at 48 h postinfection and then necropsied. Macroscopic lesion scores for the air sacs and combined lesion scores for the internal organs (heart/pericardium and liver) were determined based on a scheme similar to that used by Mellata et al. (11). The left lung, liver, and spleen of each animal were weighed, suspended in phosphate-buffered saline, and homogenized with an Omnimixer homogenizer. Dilutions of homogenates were plated onto MacConkey agar plates for bacterial quantification. The results were represented as the CFU/mL of blood or CFU/g of tissue for each strain. In the case of coinfection models, bacterial quantification on MacConkey agar plate allowed direct comparison of the virulent mutant CH138 Δ*lac* strain (white colonies) and its derivative mutants (red colonies). Competitive indices (CI) were determined for each sample and normalized for the input ratio of the inoculum. The log CI values were used for graphical representation, with negative log CI values indicating a decreased capacity of the mutant to compete with the virulent APEC strain CH138.

### Statistical analysis

All the statistical analyses were performed using GraphPad Prism 10 software (GraphPad Software, San Diego, CA, USA). Statistically significant differences between two groups were established by an unpaired *t*-test, and comparisons among three or more groups were done by a non-parametric Kruskal-Wallis test or one-way ANOVA (*P ≤ 0.05, **P ≤ 0.01, ***P ≤ 0.001, ****P ≤ 0.0001). For single-strain infections, comparisons of the CFU/mL of blood or CFU/g of tissue distributions were analyzed using the non-parametric Kruskal-Wallis test. A Wilcoxon signed-rank test (two-tailed; *P* ≤ 0.05) was used to determine statistical significance for comparison of bacterial numbers in coinfection experiments.

## RESULTS

Extending from research demonstrating an important role for *ryfA* in UPEC strain CFT073 (15), the aim of this study was to evaluate the effects of the loss of RyfA, the adjacent region encoding the TimR sRNA, or the cumulative loss of both of these sRNAs for virulence and other phenotypes in APEC strain CH138. To elucidate the role of both small RNAs, we have generated mutants wherein the DNA regions encoding *ryfA*, *timR,* or both sRNAs (*ryfA* and *timR*) have been deleted by using lambda red recombinase-mediated allelic exchange. We found that none of the deletions had any appreciable effect on the *in vitro* growth of strains in LB broth (Fig. S1).

### Loss of *ryfA* impairs type 1 fimbriae production and biofilm formation while enhancing motility

**Fig 1:**
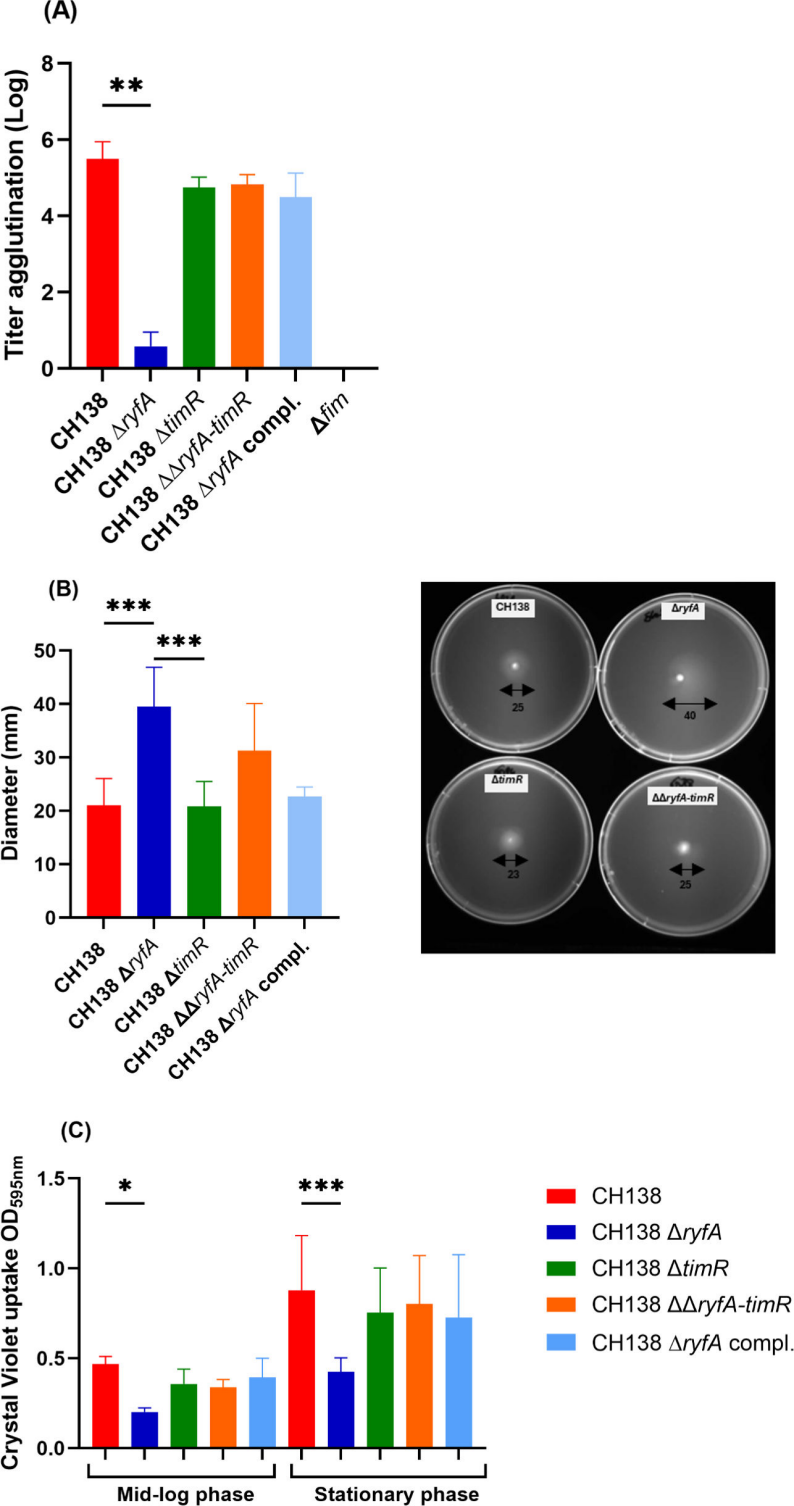
Effect of RyfA-TimR on type 1 fimbria production, motility, and biofilm formation. Production of type 1 fimbriae mediated by yeast agglutination by *E. coli* CH138 and derivative strains. (**A**) Production of type 1 fimbriae was assessed by yeast agglutination titer in strains cultured to the mid-log phase of growth in LB broth. The Δ*fim* strain was used as a negative control and showed no agglutination. (**B**) Motility of CH138, *ryfA* mutant, and complemented strain on 0.25% soft agar after incubation at 37°C for 18 h. Each box and scatter dot plot (min–max) represents the mean diameter of the motility zone. (**C**) Quantification of biofilm production by APEC strain CH138 and isogenic mutants growing in LB broth as determined by the adsorption of crystal violet at OD_595_. Results are the mean values and standard deviations for at least three biological experiments. Statistical significance was assessed using the Kruskal-Wallis non-parametric one-way ANOVA (**A, B, and C**): *P ≤ 0.05, **P ≤ 0.01, and ***P ≤ 0.001.

Since adhesins, such as type 1 fimbriae, are important virulence factors of *E. coli,* and deletion of *ryfA* was previously demonstrated to reduce *fim* expression and virulence of UPEC strain CFT073 in a UTI infection model (15), we speculated that deletion of *ryfA* in the APEC strain CH138 might also lead to decreased production of type 1 fimbriae. Cells expressing type 1 fimbriae are known to mediate yeast agglutination due to the abundance of mannans on the yeast surface, which consist of mannose units. The FimH adhesin specifically binds to D-mannose, which acts as its receptor (39, 45). To determine type 1 fimbria production mediated by yeast agglutination, wild-type strains and their derivative mutants were grown until the exponential phase (OD_600_ ≈ 0.6) in LB medium at 37°C with shaking. When comparing wild-type strain CH138 with isogenic mutants, the agglutination titer of the Δ*ryfA mutant* was significantly reduced (Fig. 1A) (*P <* 0.01). However, the level of yeast agglutination of the Δ*timR* and ΔΔ*ryfA-timR* mutants was identical to that of the parent strain (Fig. 1A). Complementation of the Δ*ryfA* mutant with a single copy of *ryfA* on the chromosome restored production of type 1 fimbriae to the level of the WT strain (Fig. 1A). Since, in some cases, a decrease in the production of type 1 fimbriae may lead to increased flagella-mediated motility (14, 46), we tested whether reduced type 1 fimbria expression influenced motility. To ensure identical conditions for type 1 fimbria production, the strains were grown in LB medium with shaking until the exponential phase. Then, the strains were inoculated onto 0.25% soft agar plates and incubated for 16–24 h ± 2 h at 37°C. After 16 h of incubation, the Δ*ryfA* strain exhibited significantly greater swimming motility on semi-solid agar plates with significantly greater diameters (*P <* 0.001) compared to the parental strain (Fig. 1B). Motility of the Δ*timR* and ΔΔ*ryfA-timR* mutants was similar to that of the wild-type parent (Fig. 1B). The same results were observed after 22 h of incubation (Fig. S2).

Since biofilm formation may also be linked to the levels of production of either type 1 fimbriae or flagella (41, 47), we next investigated and compared the biofilm-forming abilities of strain CH138 and its isogenic mutants using the crystal violet uptake assay. The Δ*ryfA* mutant displayed a significant decrease in biofilm formation when growing until mid-log phase (*P <* 0.05) or the stationary phase (*P <* 0.001) (Fig. 1C) in comparison with the wild-type strain CH138, whereas no significant difference in biofilm formation was observed for the Δ*timR* or ΔΔ*ryfA-timR* mutants. The complemented Δ*ryfA* mutant also restored biofilm formation to levels comparable to the wild-type CH138 strain (Fig. 1C). Collectively, these findings suggest that the absence of *ryfA* primarily reduces type 1 fimbria production and biofilm formation while also contributing to increased motility. Since the loss of *ryfA* in the presence of *timR* resulted in the greatest phenotypic changes, we wished to determine whether overexpression of *timR* from a multi-copy inducible plasmid in WT strain APEC CH138 could result in similar phenotypic changes. However, expression of *timR* from a plasmid in strain CH138 did not appreciably alter yeast agglutination titers, motility, or sensitivity to oxidative stress when compared to the WT background (Fig. S6).

### Loss of the *ryfA* gene increases sensitivity to oxidative and osmotic stress

**Fig 2:**
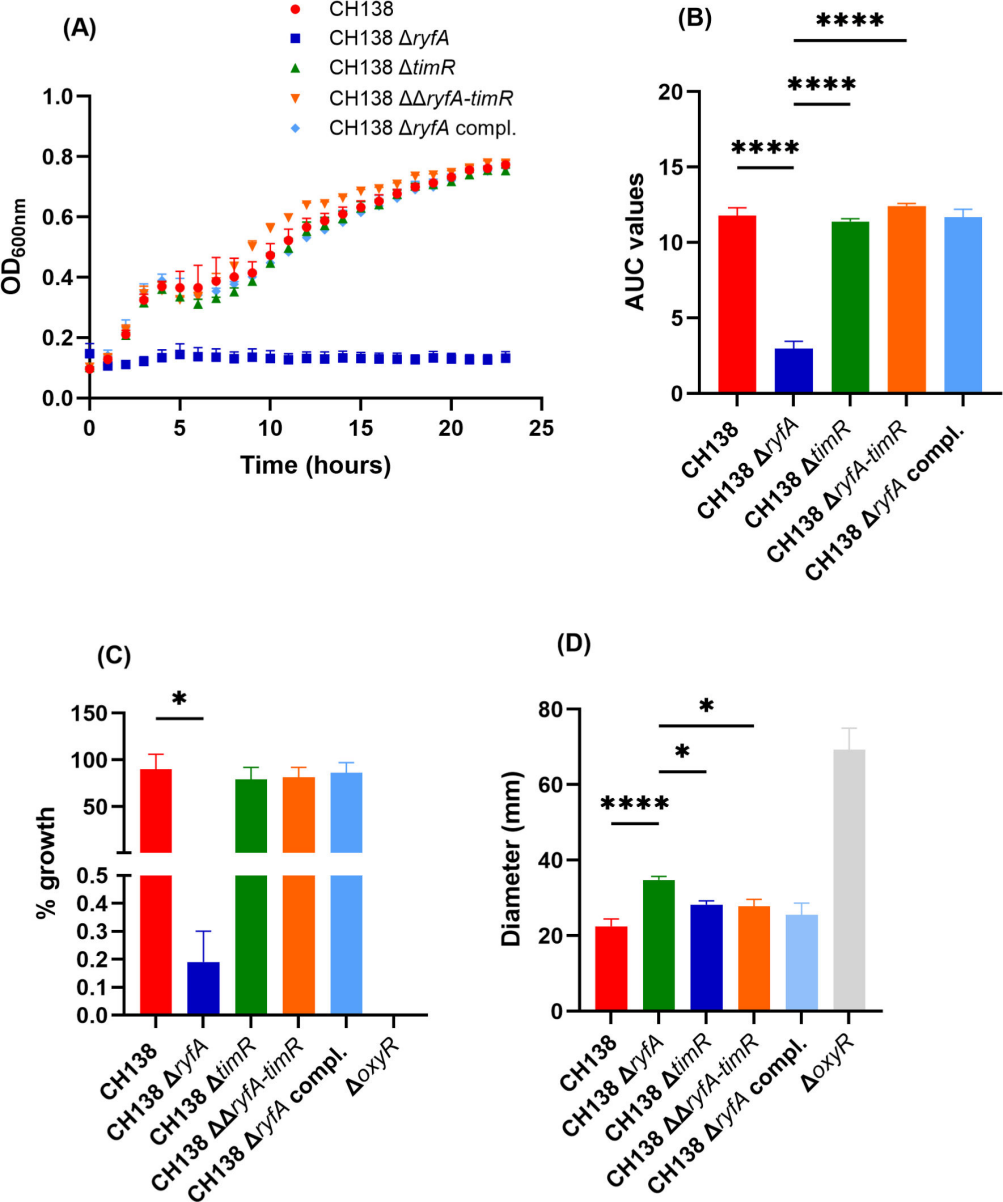
Deletion of ryfA impairs oxidative and osmotic resistance of *E. coli* APEC strain CH138. Strains were grown with shaking in LB medium until mid-log phase (O.D._600_≈0.6) for (**A**) kinetic growth and (**B**) area under the growth curve (AUC) of bacterial growth of CH138 and its derivative mutant strains; strains were inoculated in 96-well plates with LB broth supplemented with 0.6 M urea at 37°C in the Bioscreen system (Oy Growth Curves Ab Ltd., Raisio, Finland). Measurements were taken every 15 min after 10 s of shaking. Each bar represents the mean ± SEM of five independent replicates. (**C**) For the assessment of growth under 0.6 M urea osmotic stress conditions, bacteria were serially diluted with PBS and plated on LB agar supplemented with 0.6 M urea. The percentage of growth represents the surviving bacteria on LB agar supplemented with 0.6 M urea, relative to growth on LB agar plates without urea. (**D**) Growth inhibition zones (mm) on LB agar plates of CH138 and the isogenic mutants following exposure to oxidative stress induced by 50% H_2_O_2_, Δ*oxyR* was used as a sensitive control. Comparisons were made using the Kruskal-Wallis non-parametric one-way ANOVA. *P ≤ 0.05, ****P ≤ 0.0001*.*

Several studies have established a connection between oxidative and osmotic stresses and the regulation of type 1 fimbriae. In previous studies, we demonstrated that UPEC CFT073 Δ*ryfA* was significantly more sensitive to osmotic stress induced by 0.6 M urea and 0.6 M NaCl (15). The potential role of *ryfA* and the impact of the other sRNA *timR* in the general stress response were assessed in APEC strain CH138 and its derivatives (CH138 Δ*ryfA*, CH138 Δ*timR,* and CH138 ΔΔ*ryfA-timR* strains) by growth on LB agar containing urea and were compared to the growth of the wild-type strain. In the presence of 0.6M urea, compared to wild-type CH138, an evident reduction of approximately 90% was observed for survival of the CH138 Δ*ryfA* mutant (*P* < 0.0001) (Fig. 2A). By contrast, the *timR* and *ryfA-timR* double mutant grew as well as the wild-type strain (Fig. 2A). Complementation of *ryfA* also resulted in a gain in capacity to grow during exposure to 0.6 M urea (Fig. 2A). In addition, as indicated in Fig. 2C, CH138 Δ*ryfA* exhibited no detectable growth in the presence of 0.6 M urea over a 24-h kinetics assay (*P* < 0.05). In contrast, the parent strain, *timR* and *ryfA-timR* double mutant, displayed comparable and significantly higher growth. This difference was further supported by the calculated area under the curve (AUC), which revealed a significant reduction in growth for the *ryfA* mutant compared to the CH138 wild-type strain (*P* < 0.001) (Fig. 2B). By contrast, when strains were grown to stationary phase, loss of *ryfA* increased resistance to osmotic stress induced by LB 0.6 M urea (OD_600nm_≈1.2) (Fig. S3). Interestingly, under conditions where *ryfA* is more expressed (15), the mutant demonstrated improved tolerance to osmotic stress from 0.6 M urea compared to when the mutant was grown to mid-log phase (as seen in Fig. 2B).

Since RyfA’s influence on oxidative stress has been established (15), we investigated and tested the *ryfA* and *timR* mutants for resistance to H_2_O_2_. As indicated in Fig. 2D, the resistance of the *ryfA* mutant to 50% H_2_O_2_ was significantly impaired (*P* < 0.0001). Taken together, these results highlight the role of RyfA in conferring resistance to osmotic stress induced by urea and to oxidative stress caused by H_2_O_2_.

### Deletion of *ryfA* decreases resistance to chicken serum and uptake and survival of APEC in macrophages

**Fig 3:**
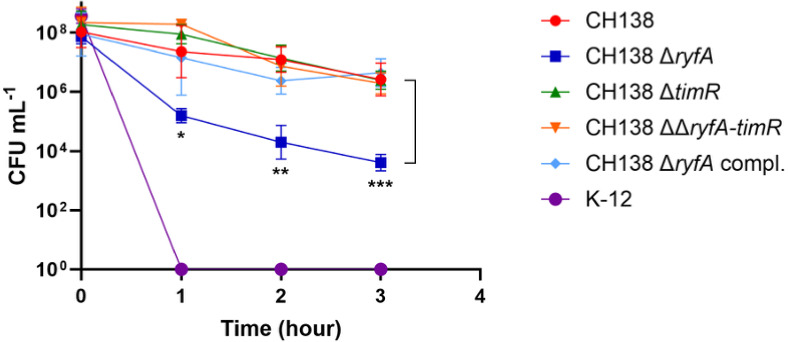
Resistance of CH138 and mutant strains to the bactericidal effect of chicken serum. Effect of 90% chicken serum on the survival of the APEC strain CH138 and its mutant derivatives and the complement strain. Bacterial cultures were grown in LB broth until mid-log phase, then incubated at 37°C in the presence of 90% chicken serum for 3 h. At designated time points (T1, T2, and T3), samples were serially diluted in PBS, and viable counts were determined by plating on LB agar plates. *E. coli* K-12 was used as a serum-sensitive control. Data are presented as mean ± standard deviation of 3–5 independent replicates. Statistical significance was assessed using a mixed-effects analysis followed by Tukey’s post hoc test. *P ≤ 0.05; **P ≤ 0.01; ***P ≤ 0.001.

To assess the role of the sRNAs RyfA and TimR in immune resistance, we evaluated the survival of the wild-type *E. coli* strain CH138 and its corresponding deletion mutants following exposure to 90% chicken serum for 3 h. Bacterial sRNAs are known to mediate responses to environmental stress, including those encountered during infection, by modulating metabolic pathways and stress responses (30, 48). A key component of the host’s innate immune defense involves activation of the complement system (49). The *ryfA* mutant strain showed significantly compromised survival in chicken serum, with a marked decrease observed after 1 h of exposure (*P* < 0.05) until 3 h (*P* < 0.001) (Fig. 3). However, the *timR* and *ryfA-timR* mutants displayed serum resistance comparable to the wild-type strain. Furthermore, the complemented *ryfA* mutant regained a serum resistance phenotype similar to that of the wild-type parent strain CH138 (Fig. 3).

**Fig 4:**
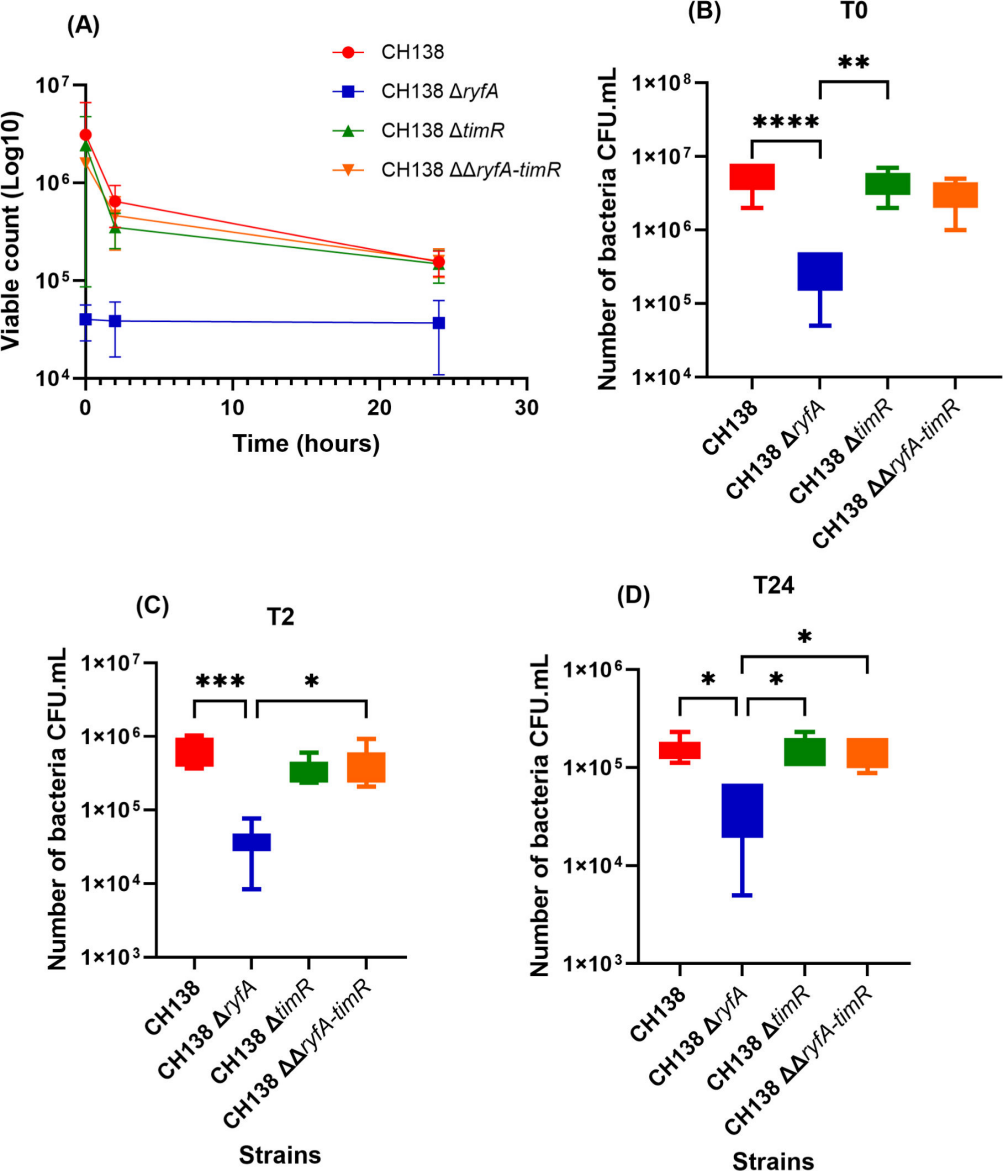
Intracellular bacterial survival assay. THP-1 cell monolayers were challenged with APEC wild-type strain CH138 or derivative mutants Δ*ryfA*, Δ*timR,* and ΔΔ*ryfA-timR* at 20:1 MOI. Cells were lysed, and intracellular bacterial counts (CFU/mL) were enumerated 2 and 24 h post-infection (pi) after extracellular bacteria were killed by treatment of cell monolayers with gentamicin at 100 µg/mL (T2) and 12 µg/mL (T24). (**A**) Survival curve of intracellular bacteria within THP-1 macrophages at different time points. Intracellular bacterial survival is compared to that of the wild-type strain at T0 (**B**), T2 (**C**), and T24 (**D**) pi. The data are represented as mean ± SEM. Statistical analysis was assessed by using the non-parametric one-way ANOVA (Kruskal-Wallis test) or one-way ANOVA *P ≤ 0.05, **P ≤ 0.01, ***P ≤ 0.001, and ****P ≤ 0.0001.

**Fig 5:**
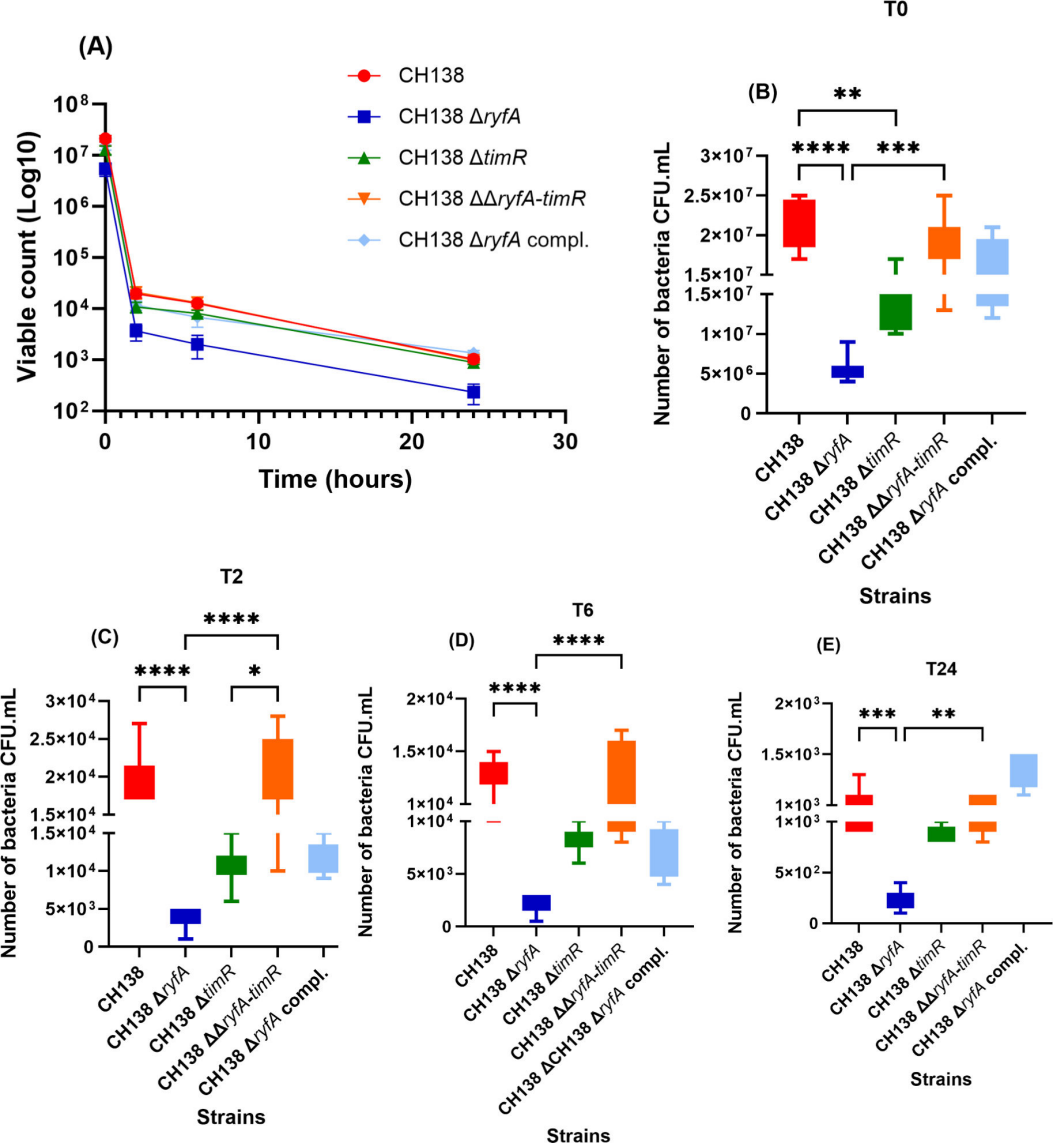
Role of RyfA during interaction with avian MQ-NCSU macrophages. MQ-NCSU avian macrophages were infected with wild-type strain CH138, Δ*ryfA*, Δ*timR,* ΔΔ*ryfA*-timR derivative mutants, or the Δ*ryfA* complemented strain. Survival curve of intracellular bacteria within MQ-NCSU cells. At different times post-infection, cells were treated with gentamicin (100 μg/mL and 12 μg/mL), and then, the cells were lysed to observe the intracellular viable bacterial count. (**A**) The initial intracellular bacterial viable counts following 1 h of interaction (T0) (CFU/mL) (**B**), then 2 h (T2) (**C**) 6 h (T6) (**D**), and 24 h (T24) later (**E**). The values are CFU per milliliter (CFU/mL) for different time points, and the results are the means of at least three experiments. Statistical analysis was performed using a non-parametric one-way ANOVA (Kruskal-Wallis test) to determine significant differences between the mutants, the wild-type strain, and the complemented mutant strains. For all analyses, *P ≤ 0.05, **P ≤ 0.01, ***P ≤ 0.001, and ****P ≤ 0.0001.

Recent studies on the sRNA *ryfA* from the UPEC strain CFT073 have demonstrated its involvement in resisting oxidative and osmotic stresses, as well as its role in survival within human primary macrophages (15). For effective extra-intestinal infection, APEC needs to disseminate in the bloodstream and must either suppress or withstand host innate immunity, including stress such as reactive oxygen and nitrogen species produced by professional phagocytes. To determine the role of *ryfA* or *timR* in the uptake and survival of APEC CH138 in macrophage cell lines, a gentamicin protection assay was performed in THP-1 and MQ-NCSU cells at an MOI of 20. The number of viable bacteria present at different times was determined by plate counting (CFU/mL). For both cell lines, the intracellular internalization of the Δ*ryfA* mutant was severely reduced in comparison to internalization of the CH138 wild-type parent strain (Fig. 4A and B) and (Fig. 5A and B) (*P* < 0.0001). However, bacterial counts for *timR* and *ryfA-timR* mutant strains did not show a significant difference with the wild-type strain in THP-1 cells (Fig. 4B). Interestingly, the *timR* mutant demonstrated a significantly reduced level of internalization in the avian MQ-NCSU cell line (Fig. 5B) (*P* < 0.01), along with a non-significant trend toward decreased bacterial persistence from T2 (*P* = 0.0519) (Fig. 5C) to T6 (*P* = 0.1233) (Fig. 5D). The intracellular survival of the *ryfA* mutant also decreased at 2 and 24 h post-infection for THP-1 (Fig. 4C and D) and MQ-NCSU cell lines (Fig. 5C through E). However, viable counts for Δ*timR and* ΔΔ*ryfA-timR* mutants were similar to those of the wild-type strain CH138 with either of these cell lines (Fig. 4B).

In accordance with previous results obtained with UPEC strain CFT073 (15), these observations also demonstrate that loss of *ryfA* alone contributes to decreased intracellular survival in macrophages. Surprisingly, at 2 h and 24 h p.i., the absence of *timR* alone did not impact intracellular survival in macrophages compared to the wild-type parent strain. Notably, the *ryfA-timR* double mutant exhibited a phenotype similar to the CH138 wild-type strain, suggesting that loss of both sRNAs may have a compensatory effect and that the significant role for *ryfA* may potentially contribute to the regulation of general stress responses through regulatory interaction with *timR.* These results highlight a key role for the *ryfA* regulatory RNA in modulating host cell interactions, contributing to uptake, survival, and replication of APEC CH138 within both human and avian macrophages. Collectively, these observations confirm a pivotal role of *ryfA* in promoting APEC CH138 resistance to serum as well as its replication and persistence within phagocytic cells. Furthermore, *timR* may play a potential role in the initial internalization of APEC CH138 by avian MQ-NCSU macrophages.

### Loss of *ryfA* attenuates the APEC strain CH138 in a chicken respiratory infection model.

**Fig 6:**
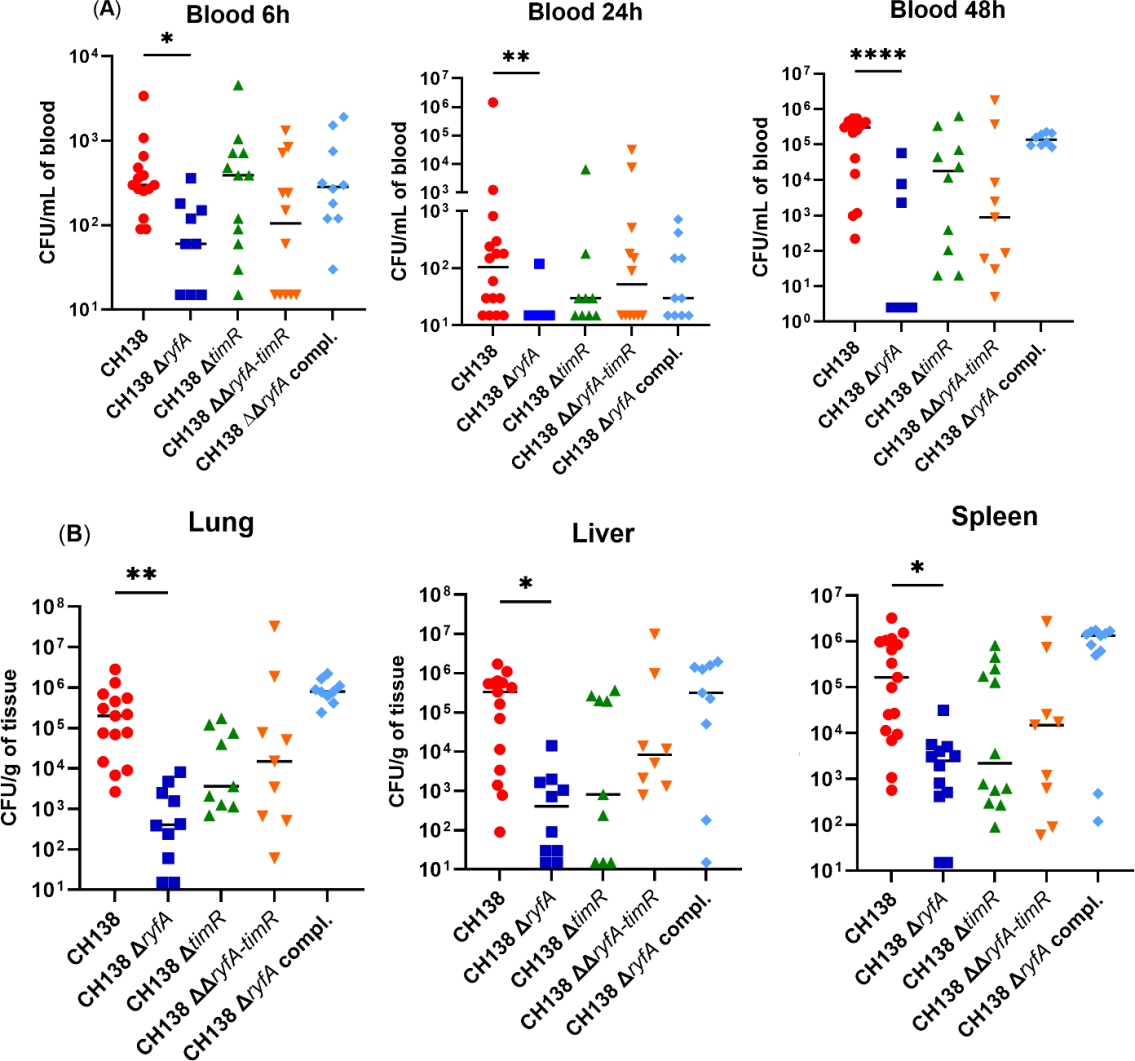
Contribution of ryfA and timR in single-strain infections in 3-week-old chickens. (**A**) APEC bacterial numbers in the bloodstream at 6, 24, and 48 hpi and (**B**) in chicken lung, liver, and spleen at 48 hpi. Each symbol represents the individual counts from a single chicken, with the median indicated by the horizontal line. Data were combined from two independent sets of experiments. Statistical analysis was performed using the Kruskal–Wallis test with uncorrected Dunn’s post-test. For all analyses, *P ≤ 0.05, **P ≤ 0.01, ***P ≤ 0.001, and ****P ≤ 0.0001.

To elucidate the role of *ryfA* and *timR* in APEC virulence during infection, we infected chickens in an air sac infection model. For the single-strain model, as previously described, chickens were infected by injection into the caudal thoracic air sac with the corresponding bacterial strains at a range of 6.20–9.45 × 10^6^ CFU. To investigate bloodstream dissemination, we assessed blood colonization by each strain at 6, 24, and 48 h post-infection (hpi). At each time point, deletion of *ryfA* severely reduced bacterial numbers in the bloodstream, with significant reductions at 6 h (*P* < 0.05), 24 h (*P* < 0.01), and 48 h post-infection (*P* < 0.0001) when compared to parent strain CH138 (Fig. 6A). Furthermore, to assess systemic infection, bacterial burden within chicken lungs, liver, and spleen was assessed at 48 hpi. The Δ*ryfA* mutant strain showed significantly compromised colonization in the lungs (*P* < 0.01), liver, and spleen (*P* < 0.05) compared to the parent strain CH138 (Fig. 6B). The Δ*timR*, ΔΔ*ryfA-timR* double mutant, and Δ*ryfA* complemented strains were present in the bloodstream and organs at levels that were not significantly different from the wild-type strain (Fig. 6A and B). Although we observed a mean decrease in lungs, spleen, and liver for the *timR* mutant, the difference was not significant compared to the wild-type parent in these tissues.

Consistent with previous findings, we postulate that the virulence of APEC strain CH138 resides in its capacity to persist and trigger a septicemic response in infected chicks, whereas the *ryfA* mutant is less able to survive and mediate a systemic infection and exhibits attenuated survival during infection.

### RyfA contributes to bacterial fitness in a competitive co-infection model with a virulent APEC strain

**Fig 7:**
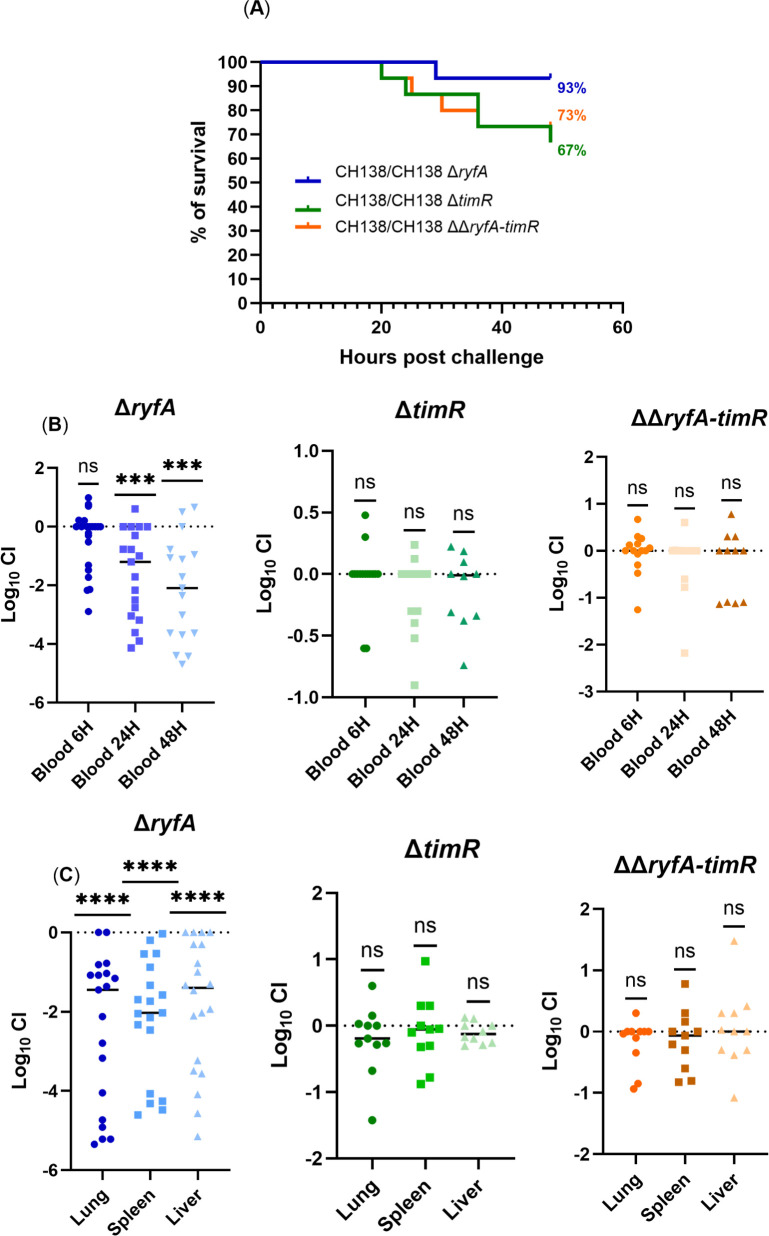
Deletion of ryfA limits colonization of chicken blood and tissues in a competitive co-infection model. (**A**) Mortality rates of co-infected groups CH138 Δ*lac*/CH138 Δ*ryfA* (blue), CH138 Δ*lac*/CH138 Δ*timR* (green), and CH138 Δ*lac*/CH138 Δ*ryfA-timR* (orange), in a chicken colibacillosis model, with survival rates determined at 48 hpi. (**B and C**) Chickens were coinfected with a 1:1 ratio of CH138 Δ*lac* and either Δ*ryfA,* Δ*timR,* or Δ*ryfA*Δ*timR* mutants. The results are presented as the log_10_ CI (**B**) for blood and (**C**) for organs. Each data point represents a sample from an individual chicken, and the horizontal bars indicate the medians. A Wilcoxon signed-rank test (two-tailed) was used to determine statistical significance; *** *P* ≤ 0.001; ****, *P* ≤ 0.0001; ns, not significant.

To assess competitive bacterial fitness, we conducted co-infection experiments in a chicken colibacillosis model, pairing the virulent CH138 Δ*lac* strain with either the *ΔryfA*, Δ*timR,* or Δ*ryfA*Δ*timR* double mutant. Co-infection models will allow a direct comparison of the virulence or fitness between two strains in the same host environment, revealing more subtle phenotypes that may not be apparent in comparative single-strain infection experiments due to inter-host variability. In line with the single-strain infection results, the Δ*ryfA* mutant was significantly outcompeted by from 200-fold to 400-fold, respectively, in lungs, spleens, and livers (*P <* 0.0001) (Fig. 7C). In contrast, Δ*timR* and ΔΔ*ryfA-timR* strains were present at similar bacterial numbers compared to the CH138 Δ*lac* strain (Fig. 7B and C). Regarding bloodstream dissemination, at the early time point of 6 h in blood, there was no competitive difference between the *ryfA* mutant and the virulent strain (Fig. 7B). However, at later time points, 24 and 48 hpi, the Δ*ryfA* mutant exhibited significantly reduced bacterial numbers compared to the CH138 Δ*lac* strain (*P* < 0.0001) (Fig. 7B). Interestingly, survival curves revealed markedly enhanced survival in chickens co-infected with CH138 Δ*lac*/Δ*ryfA* compared to those co-infected with CH138 Δ*lac*/Δ*timR* or CH138 Δ*lac*/ΔΔ*ryfA-timR*. Notably, only 7% of chickens infected with Δ*ryfA* mutants succumbed to the infection, in contrast to 27% and 13% mortality observed with co-infections with Δ*timR* and ΔΔ*ryfA-timR*, respectively (Fig. 7A). This may be due to the virulence capacity of these mutants observed in the single-strain infection studies. Altogether, these findings suggest that the loss of *ryfA*, together with the presence of the *timR* RNA, leads to attenuated virulence and increased sensitivity to multiple types of stresses. Moreover, these results highlight the essential role of RyfA in APEC colonization *in vivo*, indicating that deletion of the *ryfA* gene may compromise the capacity of an APEC strain to colonize some tissues or evade immune defenses, leading to decreased systemic infection and significantly reduced bloodstream infection and lower bacterial numbers in the lung, spleen, and liver.

## DISCUSSION

In recent years, APEC has been increasingly recognized as an extraintestinal foodborne pathogen closely related to ExPEC isolates responsible for human infections. The emergence of multidrug-resistant ExPEC strains further complicates the situation. The potential for cross-host transmission is a genuine concern, as it can occur through contact with poultry meat and eggs contaminated with APEC (3, 9, 10, 50). In this context, gaining a deeper understanding of APEC and ExPEC infections more broadly is crucial for the effective prevention and control of colibacillosis.

Bacteria are constantly exposed to a wide array of environmental stresses due to fluctuations in temperature, pH, solute concentrations, nutrient availability, and oxygen levels. During infection, bacteria must adapt and survive under hostile conditions by responding to a wide range of environmental cues through complex regulatory mechanisms that sense environmental changes and trigger coordinated responses at both the transcriptional and post-transcriptional levels. RNAs are emerging as key regulators that facilitate bacterial adaptation by regulating metabolism and expression of genes contributing to virulence. Among the RNA-based regulatory elements involved in pathogenesis are riboswitches, 5′-untranslated regions of mRNAs (5′ UTR), and small noncoding RNAs (sRNAs) (30, 51). Approximately 90 sRNAs have been identified in *E. coli;* many of them are known to contribute to bacterial virulence (52). Evidence of sRNA involvement in the regulation of pathogenic processes is supported by several studies showing that sRNAs expressed in various bacterial species can influence responses to environmental cues. For example, RyhB in UPEC is induced under iron starvation conditions and regulates iron homeostasis (53, 54), while GadY contributes to acid stress resistance by responding to pH fluctuations (55). Additionally, MicA in *Salmonella* Typhimurium plays a role in regulating genes involved in biofilm formation either directly or indirectly (56, 57).

A transposon (Tn) library in the UPEC reference strain CFT073 was previously generated. The library was exposed to environmental stress conditions and screened to identify mutants with reduced tolerance to stress, in order to identify genes that may potentially be involved in UPEC stress adaptation. One mutant that displayed reduced stress tolerance harbored a transposon insertion located adjacent to the small RNA, *ryfA* (16).

sRNAs can mediate crosstalk between different regulons, modulate their activity, and establish hierarchical regulation of gene expression. While the critical role of the sRNA *ryfA* as a potential environmental stress regulator has been established in UPEC strain CFT073 (15), the intricate interplay between *ryfA* and *timR* (34), a regulatory RNA located adjacent to *ryfA*, remains unclear and requires further investigation. Interestingly, the expression of *timR* has been detected in multiple strains. This includes detection by northern blot in the *S. enterica* SL1344 strain (34, 58) and the UPEC CFT073 strain (Fig. S4), and TimR expression was also confirmed in APEC CH138 strain (Fig. S5). These observations support the widespread occurrence and expression of this sRNA. In this current study, using an APEC model, we present the first functional characterization of TimR in *E. coli* and show that *ryfA* and *timR* are functionally connected and likely co-regulate stress responses that are relevant to bacterial fitness. Our findings provide new insights into sRNA-mediated regulation of APEC virulence and adaptation to host-associated environments. Deletion of *ryfA* had a significant impact on APEC virulence, characterized by a notable reduction in type 1 fimbriae as evidenced by decreased yeast agglutination, increased motility, and impaired biofilm formation. Furthermore, the *ryfA* mutant was more sensitive to oxidative and osmotic stress and had decreased survival within macrophages. Similarly, the absence of the sRNA RyfA severely affected APEC virulence, as demonstrated by reduced colonization in chicken tissues and limited internalization and replication in macrophages. In contrast, the observed phenotypes of the CH138 Δ*timR* and CH138 ΔΔ*ryfA-timR* mutants were similar to those of the parental strain CH138. Consistent with our findings, a recent report studying the sRNA protein chaperone ProQ demonstrated that RyfA, which is regulated by ProQ, contributes to virulence traits in APEC strain FY26, including survival within macrophages and chicken infection (32). However, these authors did not investigate the potential involvement of the TimR sRNA or demonstrate a specific effect of deleting *ryfA* on the expression of type 1 fimbriae. Type 1 fimbriae have previously been reported to promote adhesion to phagocytic cells by lectin-carbohydrate interactions in APEC strains belonging to the O78 and O2 serogroups (13, 59). Considering this, it is not surprising that the deletion of *ryfA*, which significantly reduced type 1 fimbria production, also decreased uptake and survival within macrophages. However, these results raise important questions. When *ryfA* is deleted, significant phenotypes are observed; however, loss of the RyfA RNA may also result in altered regulation due to the sole presence of *timR*. Thus, it remains unclear whether the observed phenotypes are due to the absence of *ryfA* or due to altered activity of *timR* in the absence of *ryfA.* Interestingly, when both sRNAs are removed in the *ryfA-timR* double mutant, the strain exhibits phenotypes comparable to the wild-type strain across all assays tested in this study. This suggests a potential compensatory effect arising from the simultaneous absence of both sRNAs. It raises the possibility that *ryfA* and *timR* may be co-regulated or functionally interconnected, perhaps acting on shared targets that positively or negatively modulate the stress response under different conditions. A key question that arises is: “How do these two sRNAs co-regulate cellular homeostasis and why does loss of *ryfA* alone, but not of both sRNAs, result in attenuation and disruption of cellular homeostasis?” As a potential explanation, it is known that in some cases, sRNAs serve dual roles, contributing to feedback regulatory loops. This is because some sRNAs act in a stoichiometric manner, that is, the regulatory RNA is degraded along with the target mRNA it is paired with. As a result, sRNA promoters are frequently among the most tightly regulated and robust within their respective stress regulons (24, 30). It is thus possible that TimR and RyfA may play interactive but complementary regulatory roles when both RNAs are present. The presence of TimR alone, in the absence of its regulatory partner RyfA, likely results in skewed or aberrant regulation by TimR. By contrast, the loss of these two closely associated sRNAs appears to abrogate most of the stress and virulence phenotypic changes observed following the individual loss of *ryfA*.

In *S. enterica,* a potential peptide named TimP has been reported to be translated from the *ryfA* transcript (34). To disentangle the regulatory role of *ryfA* from the potential contribution of the putative TimP peptide in UPEC strain CFT073, Hicham et al*.* (15) generated variant *ryfA* alleles disrupting the predicted TimP peptide and successfully complemented a Δ*ryfA* mutant to restore virulence-associated phenotypes. The results showed that while preserving the sRNA’s structural and regulatory integrity, expression of these modified alleles fully restored the wild-type phenotype in a *ryfA* mutant, suggesting that the putative peptide was not required for *ryfA-*dependent regulation in *E. coli.* Furthermore, in studies investigating *ryfA* in *Shigella dysenteriae* (60) and in APEC strain FY26 (32), no evidence was demonstrated for the production of any putative small protein, potentially encoded by *ryfA,* under the experimental conditions tested. In *S. enterica*, the interaction between TimR and RyfA has been previously reported and characterized as RNA-RNA regulatory partners, wherein TimR specifically binds to the 5′ untranslated region (UTR) of *ryfA* mRNA, thereby preventing translation of a toxic peptide, TimP, encoded by the *ryfA* RNA. This interaction constitutes a post-transcriptional toxin-antitoxin (TA) system, with TimR acting as an essential RNA to repress toxin expression (34, 61). Although it remains to be determined whether a toxic peptide may be encoded by *ryfA* in *E. coli* strains, our work herein emphasizes that the loss of *timR* has minimal effects. This indicates that RyfA is unlikely to cause deleterious effects in the absence of its binding partner, TimR, in *E. coli.* By contrast, in the present study, using the APEC model, we demonstrated that loss of *ryfA* alone induces regulatory instability, suggesting that TimR, in the absence of its partner RyfA, may exert deleterious regulatory effects, particularly under stress or virulence-inducing conditions.

Inactivation of *ryfA* in both UPEC and APEC strains results in attenuation (15, 32). Herein, we have further shown this is also the case for the APEC strain CH138. However, in addition to establishing that RyfA may act as a general stress regulator in ExPEC, the role of TimR remains unclear and requires further investigation. Stress response can be coordinated by multiple sRNAs, which often act in concert or in parallel to fine-tune gene expression. For example, RyhB is regulated by Fur and functions to repress non-essential iron-containing proteins, thereby conserving iron by upregulating iron-sparing pathways (62, 63) and iron acquisition systems (53, 54). In contrast, FnrS is activated under anaerobic and iron-deprived conditions, where it represses genes involved in aerobic metabolism and iron storage/usage. This mechanism enables the bacteria to shift their metabolism toward anaerobic pathways, thereby conserving iron (64). These two sRNAs act under overlapping stress conditions (iron limitation and low oxygen) and collaboratively optimize bacterial metabolism and iron usage during infection or environmental stress. In line with this, our findings suggest a potential coordination between both sRNAs, RyfA and TimR, as evidenced by the phenotypes observed in the double mutant *ryfA-timR*. Notably, the effect of *timR* in avian MQ-NCSU macrophages resulted in a slight decrease for this *timR* mutant; however, the absence of *timR* generally did not result in a significant difference compared to the wild-type strain. The MQ-NCSU cell line was first employed as an *in vitro* model to investigate the interaction between Lactobacilli and the host immune system. Although these cells may not fully represent the characteristics of chicken macrophages, several studies have demonstrated that MQ-NCSU cells exhibit key features of the mononuclear phagocyte lineage and closely mimic the biology and function of chicken macrophages (43). Notably, avian air sacs lack resident immune cells and depend on the recruitment of inflammatory heterophils as the initial cellular defense, followed by the entry of macrophages (11, 13). This could explain the slight decrease in bacterial burden observed in chicken mono-infection for the *timR* mutant in internal organs, mainly in the lungs.

During APEC infection, the respiratory tract is recognized as the primary route of infection, which can lead to systemic disease. To investigate the virulence factors of APEC, various inoculation methods have been employed, among which intra-air sac administration is considered the most effective direct route. This approach ensures a standardized delivery of a defined bacterial dose directly to the site of natural infection, thereby closely mimicking the natural course of bacterial respiratory infection. Moreover, air sac infection may bypass the mucosal defenses of the upper respiratory tract, which might otherwise limit bacterial access to the lower respiratory system and consequently reduce bacterial dissemination to the bloodstream and various organs (18, 65). This route of infection may help explain the slightly reduced bacterial burden observed for the APEC *timR* mutant in the single-strain model, potentially due to the lack of resident macrophages in this site, to which the *timR* mutant appears to be sensitive. The inability of the *timR* mutant strain to establish infection could be attributed to its impaired capacity to overcome host innate immune responses, an interpretation supported by our findings of diminished bacterial replication in the MQ-NCSU avian macrophage line. However, there was no disadvantage for the *timR* mutant in the co-infection model, suggesting that the presence of the wild-type strain at a 1:1 ratio may affect the host defense response and compensate for the attenuated phenotype observed with the *timR* mutant in single-strain infection. Among the tested mutants in this study, only the deletion of *ryfA* resulted in a significant competitive disadvantage compared to *timR* and *ryfA-timR* mutants. Consequently, this finding is consistent with previous observations in UPEC CFT073, where the absence of *ryfA* similarly leads to decreased virulence (15). This attenuation may result from the impaired biofilm formation observed in this study, phenotypes previously associated with the regulatory activity of the sRNA RyfA in other strains (15, 32, 66) or from the combined effects of all observed phenotypes. As proposed, in the UPEC model, RyfA may act as a general stress regulator, modulating multiple pathways that collectively contribute to reduced virulence. However, the underlying regulatory mechanism of *ryfA,* as well as its potential co-regulatory interactions with *timR,* will need further investigation to elucidate their contribution to the general stress response in ExPEC. Importantly, TimR remains expressed in the Δ*ryfA* mutant, and the phenotype observed upon the deletion of *ryfA* may reflect an imbalance or specific regulatory activity of TimR in the absence of its regulatory partner, rather than from the loss of RyfA alone. Conversely, the complete deletion of the RyfA-TimR sRNA module resulted in a phenotype mostly similar to the wild-type strain, suggesting that the combined presence of these two RNAs is not essential under the tested conditions and that compensatory regulatory pathways may maintain cellular regulation and adaptation during infection in their absence.

### Conclusion

Given the zoonotic potential of ExPEC, APEC represents a significant public health concern. Our study provides novel insights by identifying TimR as a functional component of the RyfA regulatory network in an APEC strain and shows that RyfA and TimR together modulate stress resistance and virulence. Disruption of this sRNA pair results in distinct phenotypes depending on whether one or both sRNAs are deleted. This study provides the first evidence in an *E. coli* model that RyfA and TimR act as a coordinated regulatory complex to maintain balance in bacteria during stress adaptation and pathogenesis in ExPEC. In conclusion, our study sheds light on the regulatory roles of sRNAs in APEC virulence, particularly *ryfA*, which emerges as a critical factor in stress adaptation, biofilm formation, and immune evasion. While *timR* appears to play a more subtle or context-dependent role, its interaction with *ryfA* suggests a layered regulatory network. These findings lay the groundwork for future research into sRNA-based regulatory circuits and their potential as targets for strategies to prevent or treat infections caused by *E. coli* and possibly other related bacterial pathogens.

## Data Availability

All data relating to this article are present in the article and the accompanying supplemental material and are openly available in Borealis, the Canadian Dataverse Repository, at https://doi.org/10.5683/SP3/OYY3C0 (67).

## SUPPLEMENTAL MATERIAL

Supplemental material aem.02000-25-s0001.docx Tables S1 and S2; Fig. S1 to S7.

ASM does not own the copyrights to Supplemental Material that may be linked to, or accessed through, an article. The authors have granted ASM a non-exclusive, world-wide license to publish the Supplemental Material files. Please contact the corresponding author directly for reuse.
