# Supplementary material for: Orchestrating infection: the impact of RyfA and TimR sRNAs on stress resistance and virulence in avian pathogenic Escherichia coli in chickens
Source: Appl Environ Microbiol. 2026 Apr 29;92(5):e02000-25. doi: 10.1128/aem.02000-25 (PMC13188913; doi:10.1128/aem.02000-25)
Supplement: Supplemental material — Tables S1 and S2; Fig. S1 to S7. [file aem.02000-25-s0001.docx]

# **SUPPLEMENTAL DATA**

**Table S1.Bacterial strains and plasmids used in this study.**

| **Bacterial strains** | **Relevant genotype** | **References** |
| --- | --- | --- |
| CH138 | APEC wild-type strain (O1:K1:H7) | 35 |
| QT5605 | CH138 ΔryfA::FRT | 35 |
| QT5607 | CH138 *att*Tn7-Cm::*ryfA,* Cm^r^ | 35 |
| QT6094 | CH138 ΔtimR::FRT | This study |
| QT6088 | CH138 ΔΔryfA-timR::FRT | This study |
| QT3108 | CH138 ΔlacZYA::FRT | 12 |
| QT2799 | *Serratia liquefaciens* | ATCC 27592 |
| QT1573 | *Salmonella enterica* serovar Typhimurium SL1344 | 66 |
| QT2077 | APEC strain Δfim::FRT | 39 |
| QT4652 | *E. coli* K-12 BW25113 | 36 |
| **Plasmids** | **Relevant genotype** | **References** |
| pKD4 | Template plasmid for the amplification of the km cassette bordered by FRT sites | 36 |
| pKD46 | λ-Red recombinase plasmid Ts replicon; Ap^r^ | 36 |
| pIJ258 (pSTNSK) | pST76-K::*tnsABCD*, Km^r^ | 38 |
| pIJ360 (pSTNSK) | pGP-Tn7-FRT:: Cm, Ap^r^, Cm^r^ | 38 |
| pCP20 | FLP helper plasmid Ts replicon; Ap^r^ Cm^r^ | 36 |
| pGP-Tn7-Cm | pGP-Tn7-FRT:: Cm, Ap^r^, Cm^r^ | 38 |
| pIJ546 | pGP-Tn7-Cm::*ryfA*; Ap^r^,Cm^r^ | 35 |

**Table S2. Primers used in this study.**

| **Primers** | **Direction** | **Characteristics** | **Sequences 5' ➝ 3'** |
| --- | --- | --- | --- |
| CM2337 | Forward | Amplification of the Km^r^ cassette from pKD4 vector for *ryfA* deletion (used with CMD2338) | TTTTTGTCAAGCGAAA GAGAGTAATCATTGTT TATTTAGCGTATTATC GAGTGTAGGCTGGAG CTGCTTC |
| CMD2338 | Reverse |  | CTTTAAACAGAACCG GATAATCTAAAATAT GCCGCCCCAAAGGGC GGCATATGGGAATTA GCCATGGTCC |
| CMD2356 | Forward | Screening for *ryfA* mutation  (used with CMD2357) | TATTGGCATTGAAGCC GATG |
| CMD2357 | Reverse |  | GATTTACCGGTTGAGC CAGA |
| CMD2362 | Forward | Cloning of *ryfA* into pGPTn7-Cm plasmid and amplification of *ryfA* fragment from CFT073 for complementation  (used with CMD2363) | GCAAGGCCTTCGCGA GGTACCAGCAGATTT ACCGGTTGAGC |
| CMD2363 | Reverse |  | CGGGCTGCAGGAATT CCTCGAGCGTGACTTT AAACAGAACCG |
| CMD2764 | Forward | Amplification of the Km^r^ cassette from pKD4 vector for *timR* deletion  (used with CMD2765) | AAAAAGAGAGTTACTGGCGA  GTATTGCTTTGCTGTGAAGCACCTGC  GTTGGTGTAGGCTGGAGCTGCTTC |
| CMD2765 | Reverse |  | GCTTTTAACAGGCAATAAAAAACCGCC  GAATTTGGCGGTTTTTTATTGCTAT  GGGAATTAGCCATGGTCC |
| CMD2341 | Forward | Screening for *timR* and *ryfA-timR* mutations  (used with CMD2356) | TATCACGCCCATACAATCCG |
| CMD266 |  | In Km^r^ cassette for screening | CAGTCATAGCCGAATAGCCT |
| CMD26 | Forward | In *glmS* for screening integration in *att*Tn7 site (used with CMD1416) | GAT CTT CTA CAC CGT TCC GC |
| CMD1416 | Reverse | In Tn7 (Used with CMD26) | GCT TTT TCA CAGCAT AAC TGG A |
| **qRT-PCR** | | | |
| CMD392 | Forward | *rpoD* amplification  (used with CMD393) | GCGTGAAGCGAAAGTTCTGCGTAT |
| CMD393 | Reverse |  | TCGCGGGTAACGTCGAACTGTTTA |
| CMD2760 | Forward | *timR* amplification  (used with CMD2761) | GCCGAATTTGGCGGTTT |
| CMD2761 | Reverse |  | CGTTGCTCATGCAACACAAG |

**Figure S1. Growth of CH138 wild-type and its derivative mutant in LB media**

Strains were grown in LB media at 37 °C with shaking at 250 rpm until mid-logarithmic phase. Growth kinetics in LB medium were compared for each strain, and kinetic curves were generated. Bars represent the mean ± SEM from 5 independent replicates.

**Figure S2. Effect of loss of ryfA and timR on motility**

Motility of CH138, *ryfA* mutant and complemented strain on 0.25% soft agar after incubation at 37°C for 22h. The results shown are the mean values and standard deviations for four biological experiments. Statistical significance was calculated by the one-way ANOVA ****, *P* *≤* 0.0001.

**

**Figure S3. Impact of ryfA and timR on osmotic stress with urea**

Assessment of growth under 0.6M urea osmotic stress conditions. Strains were grown shaking in LB broth until the stationary phase (O.D._600_≈1.2). Bacteria were then serially diluted with PBS and then plated on LB agar with 0.6M urea. (**A**) Kinetic growth and (**B**) area under the growth curve (AUC) of bacterial growth of CH138 (red), Δ*ryfA* (blue), Δ*timR* (green) and ΔΔ*ryfA-timR* (orange) mutant strains. Each bar represents the mean ± SEM of 5 independent replicates. Comparisons were made by the non-parametric one-way ANOVA (Kruskal-Wallis), *****P ≤* 0.0001.


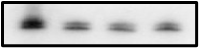


2.0

1.3

0.6

Ladder

CFT073

0.6

1.5

2.0

3.0

DO_600nm_

***timR***

100

200

300

400

500

750

1000

50

2.3

SL1344


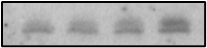


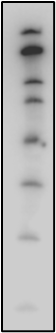


**Figure S4. Northern blot analysis of timR expression in wild-type UPEC CFT073 and S. enterica SL1344 strains.**

Total RNA was extracted from cultures grown in LB at 37 °C from mid-log to late stationary phase (O.D._600_≈0.6-3.0). For each sample, *timR* transcript (≈100nt) was detected by northern blot*.* Transcript sizes (nt) were estimated using an RNA molecular size marker.

**Figure S5. Relative expression of timR normalized to rpoD in CH138 wild-type strain**

Expression levels of *timR* in the CH138 wild-type strain were grown until mid-log phase OD_600_ 0.6 in LB at 37°C, with shaking at 250 rpm. The 2^–ΔCt^ method was used and normalized to the reference gene, *rpoD.* -RT indicates the no-reverse-transcriptase control, which did not show detectable amplification, confirming RNA specificity. Bars represent the mean ± SD from n = 2 technical replicates.

**Figure S6. Effect of overexpression of TimR on type 1 fimbriae production, motility and oxidative stress sensitivity** CH138 + ptimR was cultured in LB broth with 0.2% glucose, then the ptimR was induced with 0.1% arabinose. (**A**) Type 1 fimbriae production of type 1 fimbriae was determined by yeast agglutination titer in strains cultured to the mid-log phase of growth in LB broth. The Δfim strain was used as a negative control and showed no agglutination. (**B**) Motility in 0.25% soft agar of CH138, ΔryfA and CH138 + ptimR after incubation at 37°C for 22h. (**C**) Growth inhibition (diameter in mm) of CH138, ΔryfA and CH138 + ptimR to oxidative stress (50% H_2_O_2_) on LB agar plates. The ΔoxyR strain was used as a positive control and showed high sensitivity. Results are the mean values and standard deviations for at least three biological experiments. Statistical significance was assessed using the Kruskal-Wallis non-parametric one-way ANOVA (A, B, C): *P ≤ 0.05 and **P ≤ 0.01.


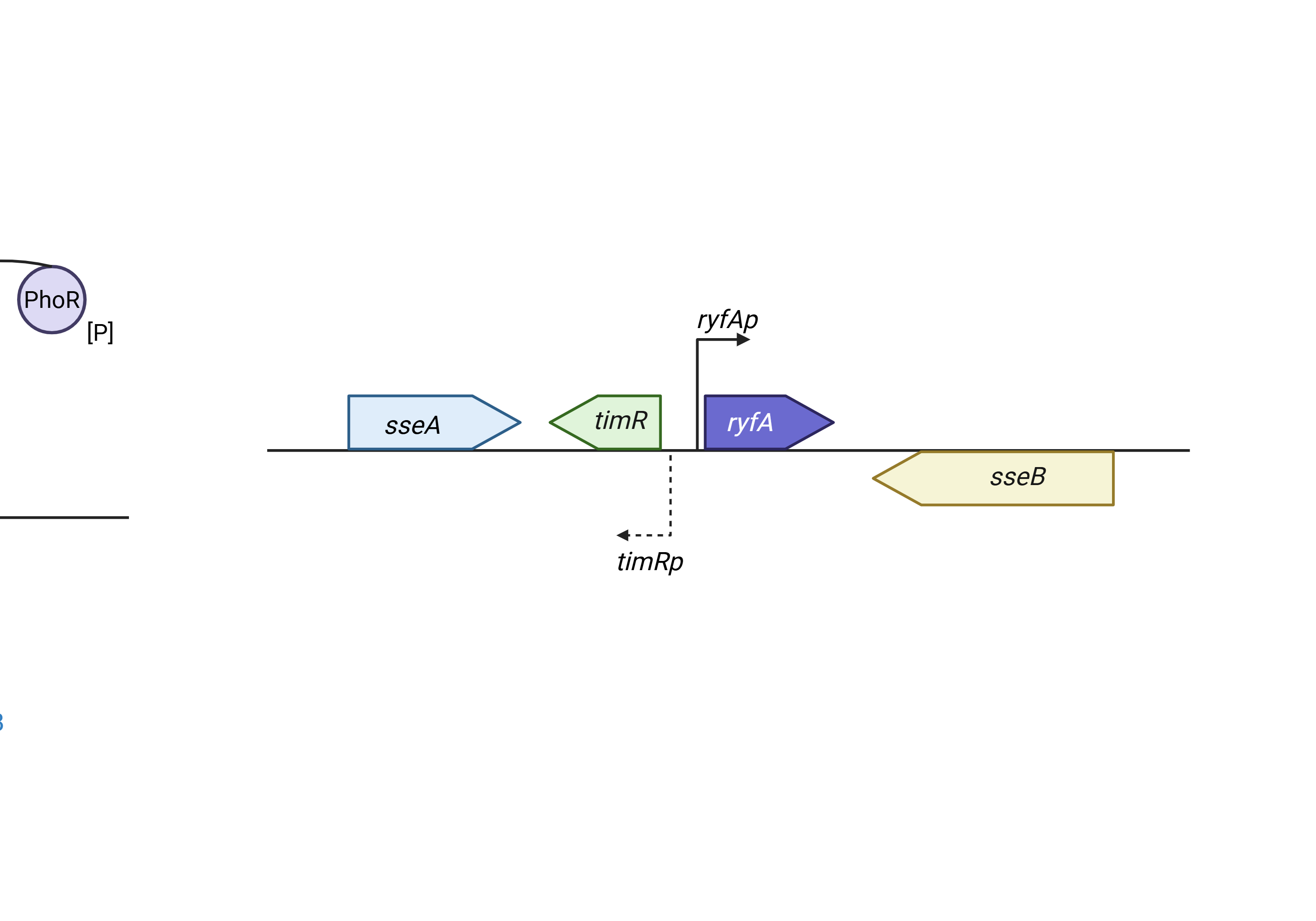


**Figure S7. Schematic representation of the genomic region of ryfA and timR in CH138**

The promoter of RyfA has been identified in *E. coli* K-12 strain but not in CH138. Putative promoters of RyfA and TimR in APEC strain CH138 are indicated by dashed arrows.
